# N-acetylcysteine improves established monocrotaline-induced pulmonary hypertension in rats

**DOI:** 10.1186/1465-9921-15-65

**Published:** 2014-06-14

**Authors:** Marie-Camille Chaumais, Benoît Ranchoux, David Montani, Peter Dorfmüller, Ly Tu, Florence Lecerf, Nicolas Raymond, Christophe Guignabert, Laura Price, Gérald Simonneau, Sylvia Cohen-Kaminsky, Marc Humbert, Frédéric Perros

**Affiliations:** 1 Univ. Paris-Sud, Faculté de Pharmacie, Châtenay Malabry, France; 2 UMRS 999, INSERM et Univ. Paris–Sud, Laboratoire d’Excellence (LabEx) en Recherche sur le Médicament et l’Innovation Thérapeutique (LERMIT), Centre Chirurgical Marie Lannelongue, 133 Avenue de la Résistance, 92350 Le Plessis Robinson, France; 3 AP-HP, Service de Pharmacie, Département Hospitalo-Universitaire (DHU) Thorax Innovation, Hôpital Antoine Béclère, Clamart, France; 4 Univ. Paris-Sud, Faculté de Médecine, Le Kremlin-Bicêtre, France; 5 AP-HP, Centre National de Référence de l’Hypertension Pumonaire Sévère, Département Hospitalo-Universitaire (DHU) Thorax Innovation, Service de Pneumologie et Réanimation Respiratoire, Hôpital Bicêtre, Le Kremlin-Bicêtre, France; 6 Department of Pulmonary Hypertension, Royal Brompton Hospital, London, England

**Keywords:** Pulmonary hypertension, Immunomodulation, Inflammation, Right heart function, N-acetylcysteine

## Abstract

**Background:**

The outcome of patients suffering from pulmonary arterial hypertension (PAH) are predominantly determined by the response of the right ventricle to the increase afterload secondary to high vascular pulmonary resistance. However, little is known about the effects of the current available or experimental PAH treatments on the heart. Recently, inflammation has been implicated in the pathophysiology of PAH. N-acetylcysteine (NAC), a well-known safe anti-oxidant drug, has immuno-modulatory and cardioprotective properties. We therefore hypothesized that NAC could reduce the severity of pulmonary hypertension (PH) in rats exposed to monocrotaline (MCT), lowering inflammation and preserving pulmonary vascular system and right heart function.

**Methods:**

Saline-treated control, MCT-exposed, MCT-exposed and NAC treated rats (day 14–28) were evaluated at day 28 following MCT for hemodynamic parameters (right ventricular systolic pressure, mean pulmonary arterial pressure and cardiac output), right ventricular hypertrophy, pulmonary vascular morphometry, lung inflammatory cells immunohistochemistry (monocyte/macrophages and dendritic cells), IL-6 expression, cardiomyocyte hypertrophy and cardiac fibrosis.

**Results:**

The treatment with NAC significantly decreased pulmonary vascular remodeling, lung inflammation, and improved total pulmonary resistance (from 0.71 ± 0.05 for MCT group to 0.50 ± 0.06 for MCT + NAC group, p < 0.05). Right ventricular function was also improved with NAC treatment associated with a significant decrease in cardiomyocyte hypertrophy (625 ± 69 *vs.* 439 ± 21 μm^2^ for MCT and MCT + NAC group respectively, p < 0.001) and heart fibrosis (14.1 ± 0.8 *vs.* 8.8 ± 0.1% for MCT and MCT + NAC group respectively, p < 0.001).

**Conclusions:**

Through its immuno-modulatory and cardioprotective properties, NAC has beneficial effect on pulmonary vascular and right heart function in experimental PH.

## Introduction

Pulmonary arterial hypertension (PAH) is a rare condition characterised by small pulmonary artery remodeling, leading to chronic pre-capillary pulmonary hypertension (PH) (mean pulmonary artery pressure above 25 mmHg and mean pulmonary artery wedge pressure below 15 mmHg), elevated pulmonary vascular resistance and right heart failure [1]. Unfortunately, despite medical treatments, progression of disease leads to right heart dysfunction, low cardiac output (CO), progressive decline in exercise capacity and eventually the development of end-organ insufficiency [1]. In addition to vasoconstriction, remodeling and thrombosis, inflammatory mechanisms play a key role in both human and experimental PH [2-8]. Pro-inflammatory cytokines including interleukin (IL)-1β and IL-6 have been reported to be elevated in both human idiopathic PAH (IPAH) [9] and monocrotaline (MCT)-induced PH [10,11]. Moreover in IPAH, infiltrates of macrophages and lymphocytes were found in the range of plexiform lesions with local expression of pro-inflammatory chemokines [12-14]. Dendritic cells were also reported in vascular pulmonary lesions and suspected to be involved in pulmonary vascular remodeling [15]. Pulmonary vascular remodeling promotes elevation of pulmonary vascular resistance leading to right ventricular hypertrophy characterized by cardiomyocyte hypertrophy and extracellular matrix changes with fibrosis. Maladaptative neurohormonal signaling, oxidative stress and inflammation in the heart have been suggested as processes possibly accelerating the development of the right-heart failure in PAH [16]. Recently, oxidative stress was speculated to play a role in the pathophysiology of human and experimental PH [17-20]. Markers of oxidative stress were found in pulmonary vascular lesions of PAH patients [21] and urinary isoprostanes have recently been shown to independently correlate with survival [22]. Inflammation and oxidative stress are thus two interlinked pathophysiological processes promoting a vicious circle in PAH pathophysiology [23].

N-acetylcysteine (NAC) is an amino acid derived from cysteine with anti-inflammatory and antioxidant properties already used in the clinical setting, for example in acetaminophen intoxication, idiopathic pulmonary fibrosis, bronchitis, ischemia-reperfusion injury, cardiac injury and doxorubicin cardiotoxicity [24]. It is both an analogue and a precursor of intracellular glutathione synthesis leading to restoration of the cell redox status. NAC is also known to inhibit pro-inflammatory cytokine TNF-α and IL-1β production and NF-κB activation, to decrease the number of inflammatory cells in the lung of rats in lung injury models and to impair chemotaxis of polymorphonuclear leukocytes and monocytes [25,26]. Finally, NAC has been reported to provide cardiac protection in animal models through its antioxidant and anti-inflammatory properties [27,28]. Acting on oxidative stress and inflammation, NAC has therefore immuno-modulatory properties associated with cardioprotective effects, which could be beneficial in the management of PAH.

In this study, we hypothesized that NAC could reduce the severity of MCT-induced PH through an immunomodulatory and cardioprotective process preserving pulmonary vascular system and right heart function.

## Material and methods

### Study design

Experiments were conducted according to the European Union regulations (Directive 86/609 EEC) for animal experiments and complied with our institution's guidelines for animal care and handling. The animal facility is licensed by the French Ministry of Agriculture (agreement N° B92-019-01). This study was approved by the Committee on the Ethics of Animal Experiments CEEA26 CAPSud. All animal experiments were supervised by Dr. Frederic Perros (agreement delivered by the French Ministry of Agriculture for animal experiment N° A92–392). All efforts were made to minimize animal suffering.

Male Sprage-Dawley rats (220–250 g) were maintained in a temperature-controlled room with a 12:12 light–dark cycle and randomly divided into 1) Saline-treated control group (n = 15); 2) MCT-exposed group (n = 15) and 3) MCT-exposed group and treated with 500 mg/Kg/d of NAC from day 14 to day 28 by daily gavage (n = 15). MCT administration was performed with a single subcutaneous injection (60 mg/kg) dissolved in 1 N HCl and neutralized with 1 N NaOH. NAC and MCT were purchased form Sigma-Aldrich (France, Lyon).

### Hemodynamics

On day 28, right ventricular systolic pressure (RVSP), mean pulmonary arterial pressure (mPAP) and cardiac output (CO) were recorded. Rats were anesthetized with 35 mg/kg ketamine, 4 mg/kg xylasine and 0.5 mg/kg acepromazine. A 3.5 French umbilical vessel catheter (Tyco, Plaisir, France), angled to 90° over the distal 1 cm and curved slightly at the tip, was introduced into the right external jugular vein. With the angle directed anteriorly, the catheter was inserted 2.5 cm proximally, which placed the catheter in the right atrium. The catheter was rotated 90° counterclockwise and inserted 1.0 cm further, which placed the catheter in the right ventricle, and when advanced an additional 1.5 cm, in the pulmonary artery. CO was measure by thermodilution: A thermic probe was placed into left carotid artery and 0.25 mL of NaCl at 0°C was injected from the right external jugular vein. Hemodynamic and CO values were automatically calculated by the physiological data acquisition Cardiomax III (Phymep, Paris, France). Total pulmonary resistance (TPR) was calculated by the ratio of mPAP/CO. Systemic pressure was measured with a 25G catheter introduced in the left carotid artery.

### Tissue preparation

After exsanguination *via* the abdominal aorta, lungs were flushed with NaCl at 37°C in order to remove circulating cells. For each animal, the left lung was distended by infusion of OCT diluted in phosphate buffered saline (PBS) (1:1) into the trachea to preserve lung morphology, quick-frozen in isopentane on dry ice and stored at -80°C. Each lobe from the right lung was dissected and snap frozen in liquid nitrogen for molecular experiments.

Right ventricular hypertrophy was measured by Fulton’s index. The right ventricle (RV) was dissected from the left ventricle plus septum (LV + S), and these dissected samples were weighed to obtain the right ventricle-to-left ventricle plus septum ratio [RV/(LV + S)].

### Pulmonary vascular morphometry

Pulmonary vascular remodeling was measured by degree of occlusion of capillary arteries on 7 μm-thick sections of frozen lung tissue. Slices were fixed with acetone for 10 minutes at room temperature and then saturated with human (10%) and donkey (10%) sera in PBS for 1 hour at room temperature. We used mouse anti alpha Smooth Muscle Actin (α-SMA)-FITC from Sigma-Aldrich (clone 1A4, dilution 1/100) and rabbit anti von Willebrand Factor (Dako, dilution 1/100). Primary antibodies were incubated overnight at 4°C. Antibody binding was detected with secondary donkey anti-rabbit-Cy3 (1/100) from Jackson ImmunoResearch. One lung section per rat was analyzed (n = 14 rats per group) and all capillary arteries were classified in 4 categories: not muscularized, partially muscularized, fully muscularized and completely occluded according the presence or not of SMC-actin staining around vWF + precapillary arteries (<50 μm).

### Right ventricular histology

Seven μm-thick sections of frozen RV were stained by hematoxylin-eosin or Sirius red for cardiomyocyte circumference and percentage of collagen area analysis respectively. Cardiomyocyte circumference was measured on transversely cut myocardial fibers and was traced on the cellular border on photomicrograph of 60 cardiomyocytes (20 cardiomyocytes per field, 3 fields per slice) from 3 to 4 rats per group with a computer-assisted image-analysis system (NIS-Element BR 2.30). Photomicrographs of transverse sections of the heart stained with Sirius red were taken to measure collagen content of the heart using ImageJ software®. The percentage of collagen area was calculated on 20 fields per slice for each rat dividing the Sirius red-stained area by the total RV tissue area.

### Gene quantification by quantitative real-time reverse transcription polymerase chain reaction (RT-qPCR)

RNA was extracted from the right lungs of rats with the total RNA isolation Mini kit (Agilent technologies, France) and then eluted from silicate columns and reverse-transcribed using Omniscript Reverse Transcription kit (Qiagen, Courtaboeuf, France). Constitutively expressed β actin was selected as an internal housekeeping gene control in the comparative (2-∆∆Ct) Ct method for the relative quantification of IL-6 mRNA expression. IL-6 and β actin gene expressions were quantified by RT-PCR with TaqMan Gene Expression Assays β actin [Rn00667869_m1], IL-6 [Rn01410330_m1], and TaqMan Universal PCR Master Mix followed in an ABI Prism7000 Sequence Detection System (Applied Biosystems, Courtaboeuf, France).

### Assessment of inflammatory cells infiltration

Immunolabeling on lung 7 μm-thick sections was performed against the rat monocyte/macrophage marker ED-1 and dendritic cell marker OX62. The number of ED-1–positive cells was determined in 5 fields for each rat (n = 6 rats per group) and the number of OX62-positive cells was determined in 6 to 10 pulmonary artery adventitia for each rat (n = 3 rats per group).

### Analysis

Quantitative variables were presented as mean values ± SD. Comparisons for all parameters were analyzed by one-way analysis of variance followed by Bonferroni’s post-hoc test or a Kruskal-Wallis test followed by a Dunn’s post-hoc test for small effectives (PRISM software, GraphPad®, San Diego, LA). Statistical significance was defined as p < 0.05.

## Results

### NAC improves cardiac function and decreases pulmonary vascular remodeling in MCT-induced PH with no effect on systemic pressure

**Figure 1:**
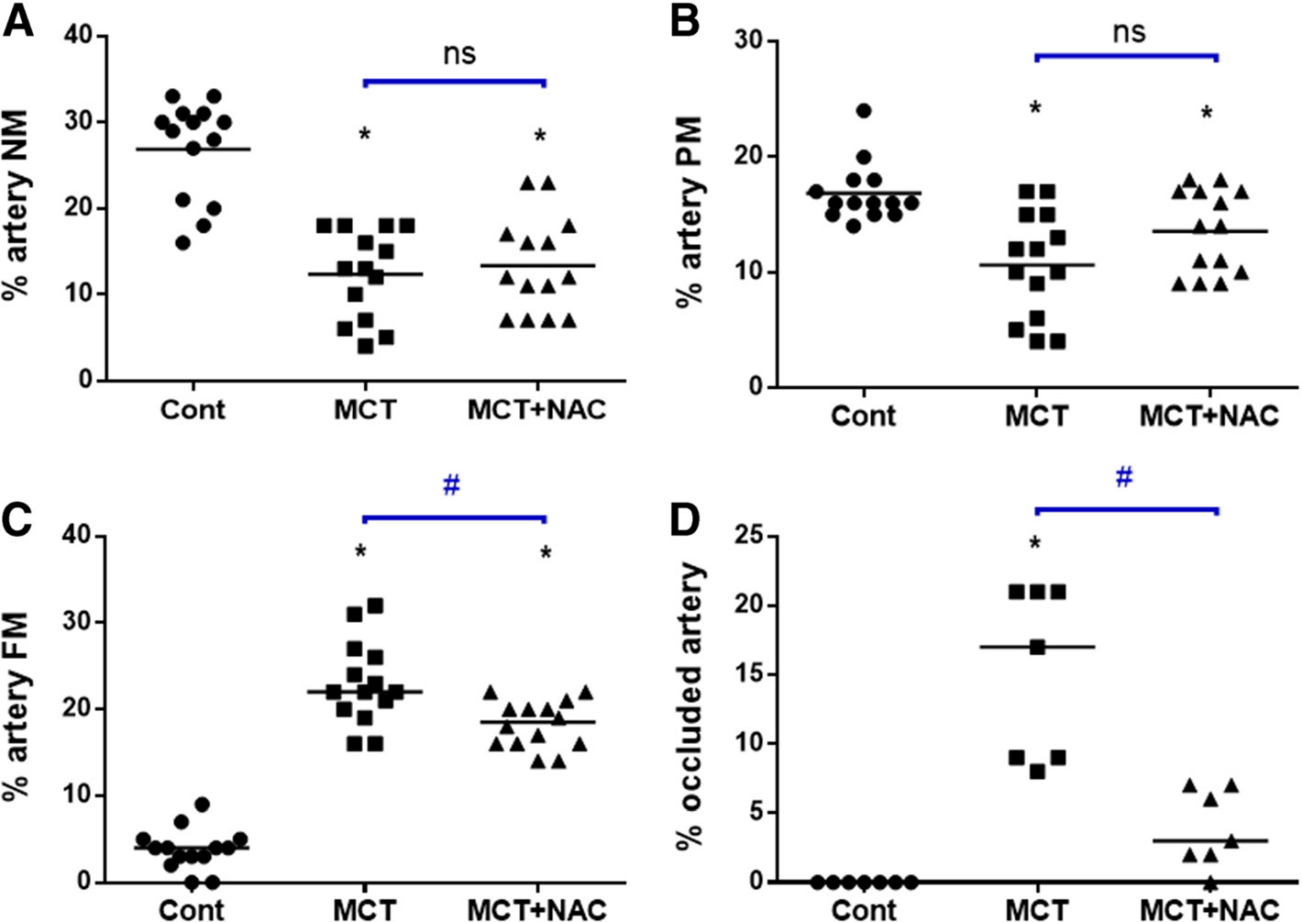
**NAC decreases MCT-induced pulmonary vascular remodeling.** Percentage of not muscularized **(A)**, partially muscularized **(B)**, fully muscularized **(C)** and completely occluded **(D)** precapillary pulmonary arteries in control, MCT and MCT + NAC groups are represented in scatter dot plot. *P < 0.05 vs cont, ^#^p < 0.05 vs MCT) (n = 7–14 per group).

Rats exposed to MCT consistently developed significant PH at day 28, with an increase of RVSP and mPAP, a fall of CO associated with high TPR. Right ventricular hypertrophy assessed by the RV/(LV+S) ratio was increased after MCT exposure due to pressure overload. Treatment with NAC from day 14 to day 28 significantly improved CO, TPR and RV hypertrophy, without effect on RVSP and mPAP (Table 1).The analysis of the pulmonary vacular remodeling quantified by the degree of occlusion of pulmonary capillary vessels highlighted a significant increase in the muscularization of small precapillary pulmonary arteries after MCT exposure, associated with a decrease in percentage of low-resistance non-muscularized vessels. Treatment with NAC significantly decreased muscularization of pulmonary capillary vessels regarding occluded and fully muscularized ones (Figure 1). On the other hand, systemic pressure measured in each group showed no differences (140.3 ± 10.6, 123.8 ± 18.3 and 126.7 ± 18.4 for control, MCT and MCT + NAC groups respectively) as well as heart rate (data not shown) excluding a peripheral beneficial impact of NAC.

**Table 1:** Hemodynamic data

	**Cont**	**MCT**	**MCT + NAC**
**RVSP (mmHg)**	38.4 ± 6.0	102.6 ± 12.1*	93.1 ± 24.7*
**mPAP (mmHg)**	14.1 ± 1.6	34.6 ± 5.8*	36.6 ± 12.2*
**CO (mL.min**^**-1**^**)**	116.6 ± 14.6	45.1 ± 4.8*	80.4 ± 21.2*^#^
**TPR (mmHg.min.mL**^**-1**^**)**	0.12 ± 0.02	0.71 ± 0.14*	0.50 ± 0.17*^#^
**RV/LV + S**	0.28 ± 0.04	0.67 ± 0.08**	0.49 ± 0.07**^##^

NAC improves MCT-induced cardiac dysfunction without effect on right ventricle and pulmonary artery pressure. Data are represented as mean ± SD. RVSP: Right ventricular systolic pressure, mPAP: mean pulmonary arterial pressure, CO: cadiac output, TPR: total pulmonary resistances. *p < 0.05 *vs* cont, **p < 0.001 *vs* cont, ^#^p < 0.05 *vs* MCT, ^##^p < 0.001 *vs* MCT (n = 8–14).

### NAC attenuates inflammatory gene expression and pulmonary infiltration of inflammatory cells

**Figure 2:**
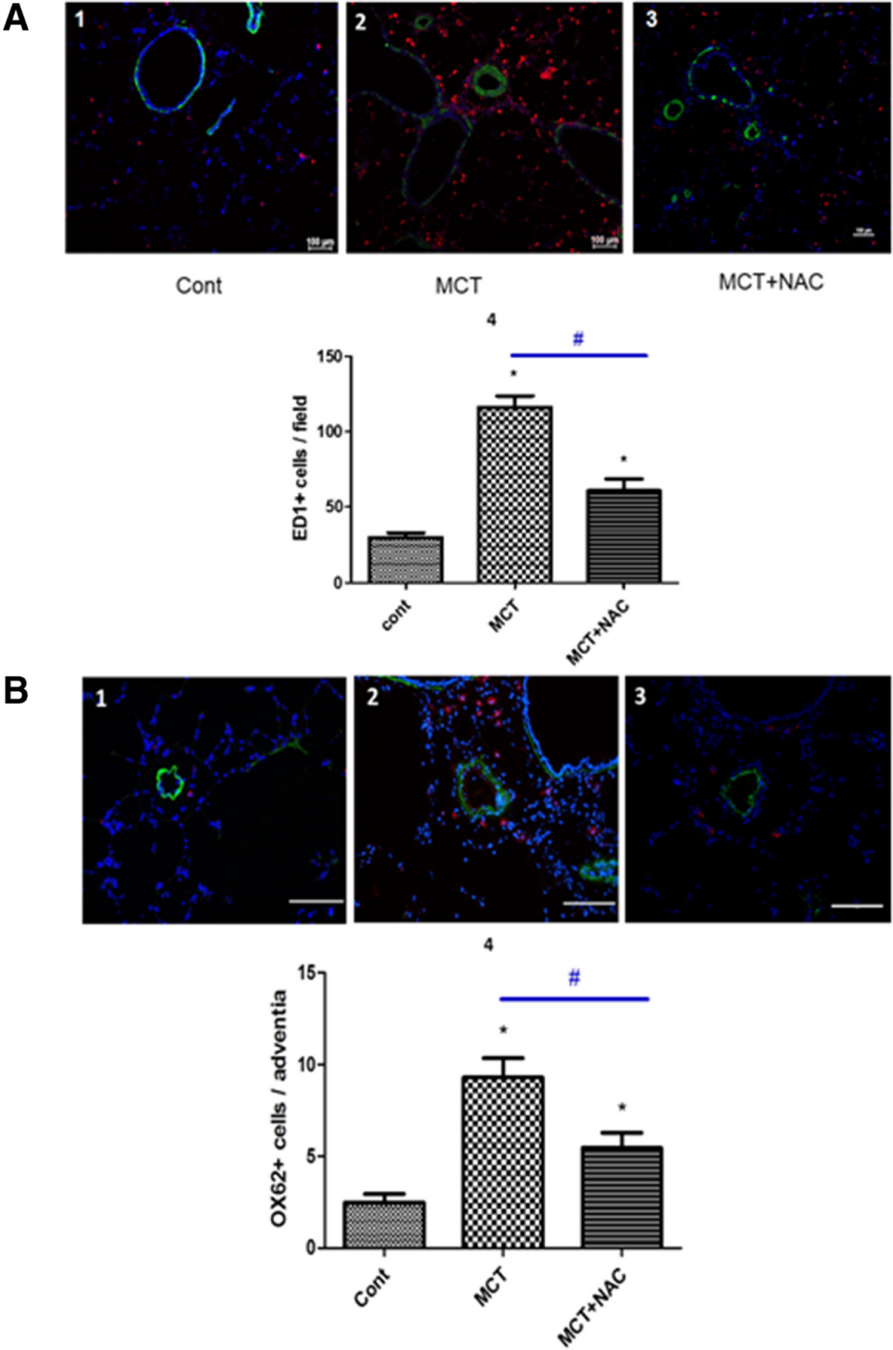
**NAC reduces monocrotaline-induced pulmonary accumulation of ED-1 and OX-62 positive cells.** Representative fluorescent images compare the presence of **(A)** ED-1 positive monocytes/macrophages (red fluorescence arrows) in control rat (1) and lung of MCT-treated rats (2). NAC treatment substantially reduced the number of ED-1 positive cells in the lungs of MCT-treated rats (3). Immunofluorescent labelling for α-smooth muscle actin (green) was used to indentify vascular smooth muscle cells (blue fluorescence: nuclei). Presence of **(B)** dendritic cells OX62 positive leucocytes (red fluorescence arrows) in lung of control rat (1) and MCT-treated rats (2). NAC treatment substantially reduced the number of dendritic cells in the lungs of MCT-treated rats (3). 4: Bar graphs are summary data for mean number of ED-1+ cells/field and OX62+ cells/adventitia (mean ± SEM). *P < 0.05 vs cont, ^#^p < 0.05 vs MCT) (n = 20–30 per group).

Development of PH in MCT-exposed rats was associated with upregulation of pro-inflammatory IL-6 mRNA expression in total lung. Treatment with NAC induced a significant decrease of IL-6 mRNA expression (0.36 ± 0.14, 2.40 ± 0.50, 1.18 ± 0.62 for control, MCT and MCT + NAC groups respectively p < 0.05). In the lungs of MCT-treated rats, the number of ED-1–positive macrophages was significantly increased compared to control group (116 ± 8 *vs.* 30 ± 3, p < 0.05). NAC treatment halved lung infiltration of ED-1–positive cells in MCT-exposed rats (61 ± 8, p < 0.05) (Figure 2A). Similar results were obtained for OX-62-positive dendritic cells in the pulmonary arteriolar adventitia of rats with a significant increase in the MCT group compared to control group (9.3 ± 1.05 *vs.* 2.5 ± 0.46, p < 0.05) and an improvement after NAC treatment (5.5 ± 0.82, for MCT + NAC, p < 0.05 as compared to MCT, Figure 2B). Thus, monocytes/macrophages and dendritic cell accumulation into lung tissue is markedly attenuated by NAC treatment.

### NAC attenuates histological signs of right ventricular damage due to PH

**Figure 3:**
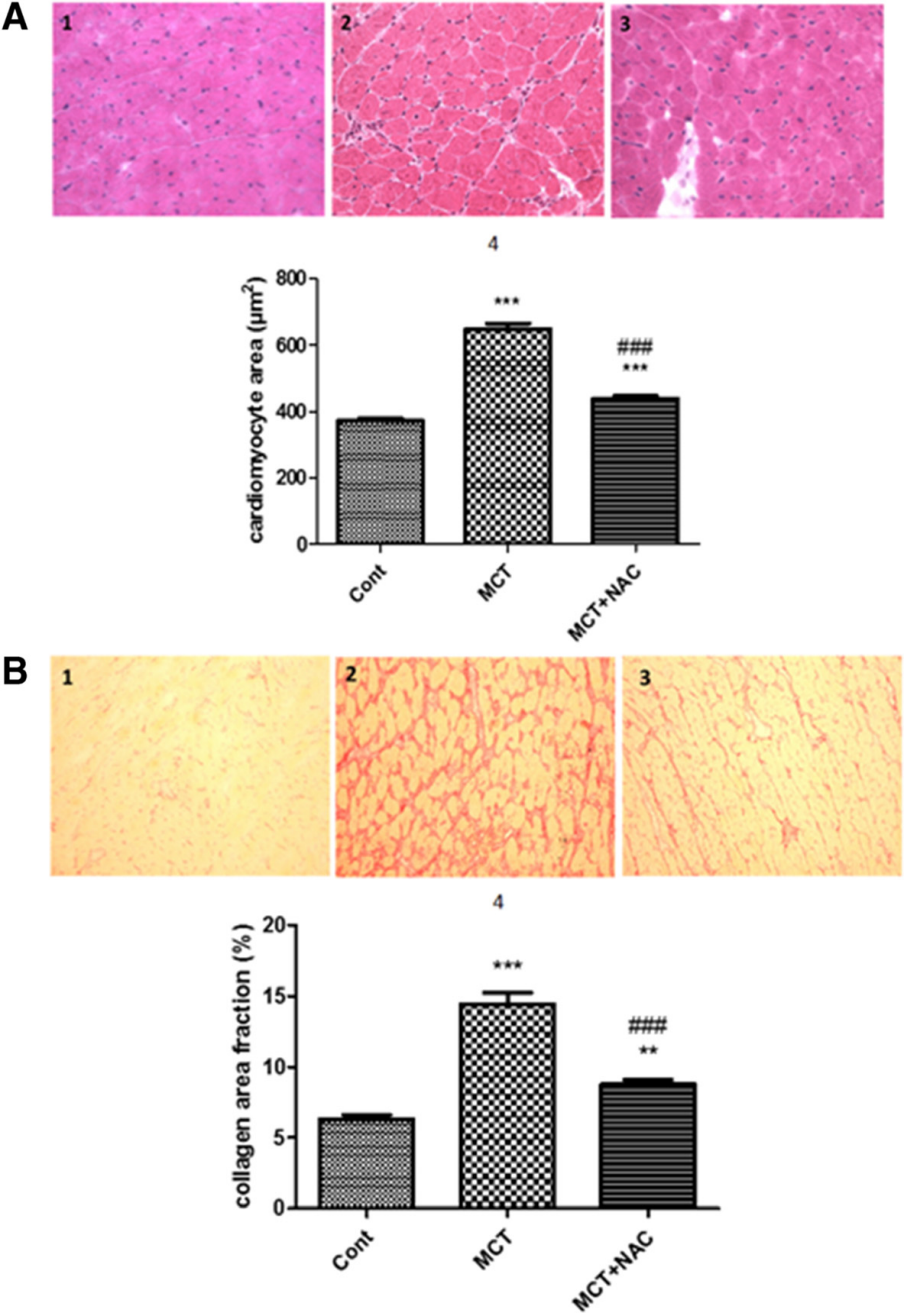
**NAC decreases cardiomyocytes hypertrophy and fibrosis in RV rats with PH.** In the first part **(A)**, representative image of cardiomyocytes in RV of control rat (1) MCT rats (2) and MCT rats treated with NAC from days 14 to day 28 after MCT exposure (3). In the second part **(B)**, representative image of collagen (red stained) content in RV of control rat (1) MCT rats (2) and MCT rats treated with NAC from days 14 to day 28 after MCT exposure (3). All photomicrographs were taken at G20 magnification. 4: Bar graphs are summary data for cardiomyocyte area (n = 180 per group) and collagen area fraction (n = 60 per group), as mean ± SEM and *P < 0.05 *vs* cont (n = 180 per group).

The circumference of RV cardiomyocytes doubled in MCT rats at 28 days compared to controls (625 ± 69 *vs.* 328 ± 46 μm^2^, p < 0.001). Treatment with NAC two weeks after MCT exposure led to decreased cardiomyocytes hypertrophy (439 ± 21 μm^2^, p < 0.001, Figure 3A). Similar results have been obtained for collagen content (fibrosis), with a significant elevation in RV of MCT exposed rats (14.1 ± 0.8 *vs.* 6.0 ± 0.7% for MCT and control group respectively, p < 0.001), and a reduction after treatment with NAC (14.1 ± 0.8 *vs.* 8.8 ± 0.1% for MCT and MCT + NAC groups respectively, p < 0.001) (Figure 3B).

## Discussion

In this study, we report that NAC reduces TPR and improves right ventricular function in rats with MCT-induced PH, partly through its immune-modulatory and cardioprotective properties. Due to the strong rationale of inflammation in PAH pathophysiology, a lot of studies have already evaluated the benefit of immune-modulatory drugs in PH animal models. However, most of them explored the drug before installation of pulmonary vascular lesions reflecting their capacity to prevent the disease and not to cure it. Despite improvement in the field of PAH, diagnosis of PAH patients is still delayed with an advanced progression of the disease, while breathlessness appears when 80% of small pulmonary arteries are affected. It is therefore essential that drugs predicted for PH improvement be studied in advanced conditions of the disease. In this study, NAC was introduced 2 weeks after MCT exposure when pulmonary vascular lesions were already developed [29], as it has been previously performed in a study using dexamethasone in the same model [30]. We choose the MCT-induced PH model for its technical simplicity and reproducibility. Although criticized by some authors, MCT remains a great model regarding inflammation process in PH [31]. It is noteworthy that other models of PH have been developed: Sugen + hypoxia- and MCT + pneumonectomy-induced PH. These models display intimal remodeling and some degree of angioproliferative lesions resembling plexiform lesions. Even if these lesions are pathognomonic of PAH (group 1 of the updated clinical classification of PH [32]), it is not in fact known whether these lesions participate to the elevated pulmonary vascular resistance responsible for PAH, or if they are just a surrogate marker of disturbed blood flow within the PAH-affected pulmonary vasculature. In contrast, the progressive neomuscularization and obstruction of precapillary resistance arteries occuring in the MCT-induced PH is a robust mechanism of TPR elevation. However it remains interesting to test NAC efficacy in other models of PAH representative of different etiologies and of potential different pathomechanisms, like chronic hypoxia or hypoxia + sugen-induced PH.

In our current study, two weeks treatment with NAC 14 days after MCT exposition led to a significant decrease of pulmonary vascular remodeling characterized hemodynamically by a reduced level of TPR and an unmodified mPAP and RVSP. In a previous study, we reported that severity of pulmonary vascular remodeling in a permanent high-flow challenged animal model associated with MCT correlated with lung expression of pro-inflammatory cytokines and recruitment of both monocyte/macrophages and dendritic cells in the pulmonary vasculature [17]. The reduction of the lung inflammatory status observed in this study with the reduced lung cytokine expression and inflammatory cells recruitment could partly explain these results. Potential disease-relevant mechanistic factors of NAC include NF-κB and Angiotensin II (Ang II) signaling. Firstly, NF-κB is the key inflammatory transcription factor well known to be activated by oxidative stress [33] which is implicated in human [21] and experimental PAH [17]; its activation leads to the upregulation of chemokines and inflammatory cytokines implicated in human IPAH [34] and in the MCT-induced PH model where its inhibition ameliorates PH - both preventatively and therapeutically [35,36]. Secondly, involvement of Ang II through its receptor 1 (AT(1)) activation was reported in the pathophysiology of PAH [37]. Interestingly, NAC decreases Ang II binding to the AT(1) receptor in vascular smooth muscle cells (VSMC) in a concentration-dependent manner, leading to a reduction of VSMC proliferation [38]. The beneficial effect of NAC could therefore be in part depends on this property. Although it has been reported evidence of NAC-induced vasodilation and hypotension [39,40], we didn’t found any systemic effect of NAC in our study.

PAH is characterized by remodeling of the pulmonary arterial vessels, which involves progressive distal vessel obliteration leading to an increase in the TPR. This increase in TPR leads to an increase in the RV afterload leading to RV dysfunction and CO decrease. In our study, mPAP was not statistically changed but TPR were decreased, the degree of distal obliteration (%) was reduced, and CO was increased in the NAC treated group. We might explain the decrease in TPR by the significant decrease in the distal artery occlusion. The simplified classical relationship between mPAP, CO and TRP is the following: mPAP = CO*TPR. In other terms, CO = mPAP/TPR. So even if mPAP remains not statistically changed, the increase in CO might be the consequence of reduced TPR and therefore of reduced distal pulmonary vascular occlusion. CO depending on proper calibration of RV contractility and impedance to blood flow through the lungs, NAC may also have a direct beneficial impact on CO though improvement of RV contractility. However, this parameter has not been analyzed in our study but deserve further investigation in order to ascertain a direct RV protective effect in PH. Another argument in favor of a direct impact of NAC on right ventricle is provided in a recent review by Voelkel on oxidative stress and PH [41]. In this review, influence of oxidative stress on pulmonary vasculature and cardiac cells was extensively analyzed, particularly in the RV failure mechanism. The authors propose a kinetic model of oxidative stress with an impact on pulmonary vasculature at an early stage and on RV at a later stage and throughout the development of the RV disease. In order to confirm their hypothesis on cardiac dysfunction, protandim treatment (having antioxidant properties) of the Su/Hx rats prevented the development of RV failure and fibrosis. Analysis of mitochondrial and metabolic gene remodeling in the RV as it was recently reported by Gomez et al., would probably also afford significant information on cardio protective pathway of NAC and need to be performed in next experiments [42].

Pressure overload usually increases pulmonary vascular resistance and cardiomyocyte stress leading to cardiomyocyte hypertrophy and extracellular matrix changes with fibrosis. Endomyocardial biopsy specimens from patients with PAH show increased levels of fibrosis affecting myocardial systolic and diastolic function [43,44]. In a recent review analyzing the RV under pressure, maladaptative neurohormonal signaling, oxidative stress and inflammation in the heart were reported as processes possibly accelerating the development of right-heart failure in PAH [16]. *In vitro* and *in vivo* studies have shown that reactive oxygen species (ROS) induce cardiomyocyte hypertrophy as well as fibrosis [45] and in the MCT-induced model of PH, right ventricular failure was associated with oxidative stress [46]. Moreover, in conditions of ischemia/reperfusion, macrophages recruit neutrophils through the secretion of IL-6, which are an important source of ROS [16]. Interestingly, Ang II induces cardiomyocyte hypertrophy, inflammation, fibrosis and contractile dysfunction through in part by the formation of ROS [47]. Due to its antioxidant, anti-inflammatory and cardioprotective properties, NAC improved right ventricular function (CO) with inhibition of cardiomyocyte hypertrophy and fibrosis.

Although specific mechanisms of NAC on RV preservation still remain elusive, improvement of right ventricular function with NAC is a particularly relevant issue since despite PAH specific therapeutics, pulmonary microvascular obstruction usually progresses and imposes an increasing load on the RV [48]. The patient outcome is therefore predominantly determined by the response of the RV to the increased afterload and RV function is therefore a strong marker of prognosis and disease severity [49]. Advance in new therapies acting on right ventricular function is therefore relevant in PAH management. However, current available or experimental PAH treatments are used to induce pulmonary vasodilation and reverse pulmonary vascular remodeling, and little is known about their effect on the heart. An ideal PAH treatment strategy would therefore both reduce pulmonary vascular resistance and improve right ventricular function. Here, we report that NAC, a well-known safe drug in current clinical use, has beneficial effect on these parameters in an experimental model of PH.

## Conclusions

In conclusion, NAC could be a potential additive treatment in PAH management preserving hemodynamic and right heart function. Further experimental and preclinical studies are needed to confirm these benefit effect.

## Abbreviations

Ang II: Angiotensin II; CO: Cardiac output; Cont: Control; MCT: Monocrotaline; mPAP: Mean pulmonary arterial pressure; NAC: N-acetylcysteine; OS: Oxidative stress; TPR: Total pulmonary resistances; RV: Right ventricle; RVSP: Right ventricular systolic pressure; SD: Standard deviation; PAH: Pulmonary arterial hypertension; PH: Pulmonary hypertension; ROS: Reactive oxygen species; VSMC: Vascular smooth muscle cells.

## Competing interests

The authors declare that they have no competing interests.

## Authors’ contributions

MCC, BR, DM, FP and LP drafted the manuscript. MCC, BR and FP carried out animal experiments and RT-PCR and designed the study. MCC, BR, PD, FL and NR carried out histochemistry, immunohistochemistry and immunofluorescence assays. MCC, BR and PD carried out morphometry and histomorphological analysis. MCC, LT, DM, FP and participated in the design of the study and performed the statistical analysis. SCK, CG, GS, MH, and FP helped to coordinate the study. All authors read and approved the final manuscript.

## Authors’ information

MCC is working as a pharmacist in Antoine Béclère hospital, Clamart and carry on its research in the research unit 999 of the ‘Institut National de la Santé et de la Recherche Médicale’ which focuses on fundamental research on the pathophysiology of pulmonary hypertension and is directed by MH. She also is teaching at the Faculty of Pharmacy, Châtenay Malabry.
